# Implications of canonical histone H3.1 and histone variant H3.3 in cancer

**DOI:** 10.1098/rsob.250133

**Published:** 2025-09-24

**Authors:** Peng Wu, Li Wang, Ting Wen, Qiao Yi Chen

**Affiliations:** ^1^ Department of Cell Biology and Genetics, Xi’an Jiaotong University, Xi’an, Shaanxi, People’s Republic of China; ^2^ Centre for Chromosome Biology, School of Natural Sciences, University of Galway, Galway, Ireland

**Keywords:** canonical histone, histone variant, H3.1, H3.3, cancer

## Abstract

Histones are the fundamental building blocks of chromatin and serve as pivotal regulators of gene expression. Differential expression and mutations of H3.1 and H3.3 genes have been implicated in the pathogenesis of various cancer types. Mutations in H3.3, especially lysine to methionine substitutions (K27M/K36M), are particularly prevalent. Moreover, genetic alterations such as G34R/W/V/L, as well as variations in *H3F3A* and *H3F3B* genes, have also been identified. Despite high similarity in amino acid sequences, H3.1 and H3.3 have discrete functions in cancer. In this review, we delve into the recent advances in elucidating the implications of canonical histone H3.1 and its variant H3.3 on chromatin structure and function. Additionally, we explore how potential enhancing factors such as PTEN, MLL5, GPR87 and histone chaperones influence H3.1/H3.3 function.

**Figure:**
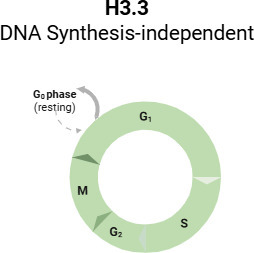

## 1. Introduction

Cancer has historically been characterized as a genetic disorder arising from dysregulation of critical cellular homeostasis pathways [1]. Malignant transformation and subsequent uncontrolled proliferation of cancer cells are driven by genetic and epigenetic alterations induced by exogenous and endogenous factors. Epigenetics encompasses heritable, reversible and dynamic processes that modulate gene expression without altering the DNA sequence [2]. Key epigenetic mechanisms include DNA and RNA modifications, histone post-translational alterations and non-coding RNA-mediated regulation [1–3]. Within this framework, histone modification represents a central pillar of epigenetic control, with profound implications for chromatin architecture and transcriptional activity [4,5]. A diverse array of histone modifications—including phosphorylation, acetylation, methylation, deamination, glycosylation and lactylation—has been shown to intricately regulate gene expression [3,5,6].

In eukaryotic nuclei, DNA is organized into chromatin through its compaction around histone octamers, forming nucleosomes as the fundamental structural units [7–9]. Histones are classified into five major families: H1, H2A, H2B, H3 and H4 [10]. The core nucleosome consists of H2A, H2B, H3 and H4, while H1 serves as the linker histone stabilizing higher order chromatin structure [11]. These proteins exhibit remarkable evolutionary conservation, with histone genes ubiquitously expressed across eukaryotic organisms [7,12,13]. Histones are broadly classified into replication-dependent (canonical) histones and replication-independent (non-canonical) variants. Canonical histones, predominantly expressed during S-phase, are synthesized in synchrony with DNA replication to support chromatin assembly in proliferating cells [2,14,15]. Notably, replication-dependent histone mRNAs are uniquely characterized by their lack of introns and polyadenylation [16]. In contrast, non-canonical histone variants—which share structural homology but diverge in amino acid sequence and expression kinetics—are incorporated into chromatin throughout the cell cycle. Their transcripts typically contain introns and poly(A) tails, reflecting distinct regulatory mechanisms [17,18]. Emerging evidence underscores the critical role of the equilibrium between canonical histones and their variant counterparts in oncogenesis. Dysregulation of this balance has been implicated in multiple cancer types, influencing chromatin plasticity, transcriptional programmes and genomic stability. This review focuses on the distinct contributions of canonical histone H3.1 and its variant H3.3 to oncogenesis and tumour progression.

## 2. Canonical histone H3.1

### 2.1. Function of canonical histone H3.1 in cancer

Nucleosome, the fundamental structural unit of chromatin, comprises a histone octamer encircled by approximately 147 base pairs of DNA. This octamer consists of two copies each of core histones H2A, H2B, H3 and H4, along with the linker histone H1 [19]. Histone H3 variants exhibit remarkable evolutionary conservation across eukaryotes [20]. Among these, the canonical histone H3.1 plays a critical role in coordinating chromatin assembly with DNA damage repair and post-damage chromatin remodelling [21]. Replication-dependent histone H3.1 is encoded by 10 genes within the HIST1 cluster on chromosome 6: *HIST1H3A*, *HIST1H3B*, *HIST1H3C*, *HIST1H3D*, *HIST1H3E*, *HIST1H3F*, *HIST1H3G*, *HIST1H3H*, *HIST1H3I* and *HIST1H3J* [22]. These genes demonstrate distinct associations with diverse malignancies. For instance, *HIST1H3A* dysregulation has been implicated in oral cavity squamous cell carcinoma, chronic myeloid leukaemia, hepatocellular carcinoma, acute myeloid leukaemia and colorectal cancer [23–25]. *HIST1H3B* alterations are linked to melanoma, colorectal cancer, diffuse intrinsic pontine glioma, medulloblastoma and metastatic histiocytic skull tumours, among others [26–31]. *HIST1H3C* is associated with lung carcinoid, neuroblastoma and tonsillar squamous cell carcinoma [32–34]. *HIST1H3D* exhibits upregulation in primary gastric cancers and lung cancer metastases, where it modulates proliferation, apoptosis and cell cycle progression, positioning it as a potential therapeutic target [10,35]. Notably, fucoxanthin—a non-provitamin A carotenoid with established anti-angiogenic and anti-tumour properties—suppresses *HIST1H3D* expression and induces apoptosis in cervical cancer cell lines [36,37]. In lung adenocarcinoma, the *HIST1H3E* locus displays pronounced differential methylation compared with normal lung tissue [38]. Upregulation of *HIST1H3F* correlates with skin, bladder and laryngeal squamous cell carcinomas [5,39,40], while *HIST1H3G* overexpression is observed in epithelial ovarian cancer and linked to DNA hypermethylation in lung adenocarcinoma and diffuse intrinsic pontine glioma [38,41]. *HIST1H3H* overexpression contributes to renal cell carcinoma, bladder cancer and tonsillar squamous cell carcinoma progression [42]. Finally, *HIST1H3J* upregulation and aberrant methylation are documented in laryngeal squamous cell carcinoma and papillary thyroid carcinoma, respectively, with prognostic relevance in oral squamous cell carcinoma [43–45].

As summarized in table 1, H3.1-encoding genes exhibit broad oncogenic associations across cancer types. However, the mechanistic underpinnings and gene-specific contributions to tumorigenesis remain poorly characterized, necessitating further investigation.

**Table 1.** H3.1 in cancer.

cancer type	H3.1 variant	up or down	references
melanoma	HIST1H3B	up	[5]
liver cancer	HIST1H3B	up	[30]
colorectal cancer	HIST1H3B	up	[30]
tonsillar squamous cell carcinoma	HIST1H3B	down	[46]
bladder cancer	HIST1H3B	up	[42]
astrocyte (cerebellum) tumours in senior adults	HIST1H3B	up	[47]
diffuse inherent pons glioma	HIST1H3B	up	[27,48]
glioblastoma in children	HIST1H3B	up	[26,48]
thalamic glioma in young people	HIST1H3B	—	[28,48]
spinal cord glioma	HIST1H3B	up	[29,48]
medulloblastoma	HIST1H3B	down	[49]
metastatic histiocytic tumour of skull	HIST1H3B	—	[31]
lung carcinoid	HIST1H3C	up	[34]
neuroblastoma	HIST1H3C	down	[32]
tonsil squamous cell carcinoma	HIST1H3C	down	[46]
primary gastric cancer	HIST1H3D	up	[10,35]
lung cancer	HIST1H3D	up	[10]
lung adenocarcinoma	HIST1H3E	up	[38]
skin squamous cell carcinoma	HIST1H3F	up	[39]
bladder carcinoma	HIST1H3F	up	[40]
laryngeal squamous cell carcinoma	HIST1H3F	up	[5,44]
epithelial ovarian cancer	HIST1H3G	up	[41]
lung adenocarcinoma	HIST1H3G	—	[38]
renal carcinoma	HIST1H3H	up	[42]
bladder cancer	HIST1H3H	up	[42]
laryngeal squamous cell carcinoma	HIST1H3J	up	[44]

### 2.2. Polyadenylation of H3.1 mRNA in malignant cell transformation

Beyond alterations in specific H3.1 genes, post-transcriptional modifications of H3.1 mRNA have been linked to malignant transformation in epithelial cells [13]. Prior studies demonstrate that arsenic induces H3.1 mRNA polyadenylation through proteasomal degradation and transcriptional repression of stem-loop binding protein (SLBP) [7,50]. This aberrant polyadenylation disrupts the homeostatic equilibrium between canonical histone H3.1 and the replication-independent variant H3.3, culminating in genomic instability and oncogenic transformation [13]. The research team revealed that arsenic-driven H3.1 polyadenylation promotes malignant phenotypes, including enhanced proliferation, anchorage-independent growth and *in vivo* tumorigenesis. Mechanistically, arsenic-induced polyadenylation caused H3.1 overaccumulation during mid-to-late S/M cell cycle phases and dysregulated cancer-associated gene expression [13]. Given H3.3’s ubiquitous cell cycle expression and structural homology with H3.1, excessive H3.1 levels competitively inhibit H3.3 incorporation into chromatin. Employing ChIP-seq, anti-FLAG affinity purification and stem-loop sequence insertion upstream of poly(A) signals, the study demonstrated that H3.1 upregulation displaces H3.3 deposition at critical regulatory elements such as active promoters, enhancers and insulator regions [13]. Crucially, ectopic H3.3 expression attenuated arsenic-induced anchorage-independent growth, underscoring the functional antagonism between these histone variants [13]. These findings suggest that arsenic-mediated epithelial cell transformation arises from H3.1–H3.3 imbalance at key oncogenic loci. Future investigations should prioritize elucidating the dynamic interplay and spatiotemporal expression patterns of H3.1 and H3.3 to unravel their context-specific roles in carcinogenesis.

### 2.3. H3.1 histone chaperones

Histone chaperones are specialized proteins that recognize, bind and orchestrate histone transport, delivery, storage and incorporation into chromatin during assembly and disassembly processes. Following histone synthesis, chaperones ensure spatiotemporal regulation of histone stability, functionality and degradation, thereby maintaining chromatin template dynamics [51]. Recent advances have identified over a dozen distinct histone chaperones, each exhibiting unique roles in nucleosome assembly and epigenetic regulation [52]. Like canonical histones and their variants, histone chaperones display structural and functional diversity that influences chromatin organization [53]. As summarized in table 2, H3.1-associated histone chaperones exhibit distinct expression profiles across malignancies. H3.1 mediates DNA synthesis-dependent nucleosome assembly primarily through the chromatin assembly factor 1 (CAF-1) complex, a heterotrimer comprising p150, p60 and p48 subunits [2,67,68]. Notably, the p60 subunit is overexpressed in prostate, cervical and hepatocellular carcinomas, as well as leukaemia and breast cancer [54–57]. Gomes *et al*. [66] demonstrated that ERK2-driven signalling downregulates CAF-1, reducing H3.1/H3.2 levels while promoting H3.3 enrichment at epithelial-mesenchymal transition (EMT)-associated gene promoters. This H3.3-mediated transcriptional reprogramming enhances metastatic potential by fostering aggressive, anchorage-independent phenotypes [69]. The interplay between histone chaperones and H3 variant dynamics not only governs chromatin architecture but also represents a therapeutic vulnerability in cancer, with CAF-1 and ERK-mediated chaperone dysregulation emerging as critical drivers of tumour invasiveness and metastatic progression.

**Table 2.** H3.1 and H3.3 histone chaperones in cancer.

chaperone	H3.1/H3.3	cancer type	up or down	references
CAF-1	H3.1	prostate cancer	up	[54]
CAF-1	H3.1	cervical cancer	up	[55]
CAF-1	H3.1	hepatocellular carcinoma	up	[56]
CAF-1	H3.1	leukaemia	up	[57]
CAF-1	H3.1	breast cancer	up	[58]
DAXX	H3.3	prostate cancer	up	[59,60]
DAXX	H3.3	ovarian cancer	up	[61]
DAXX	H3.3	oral squamous cell carcinoma	up	[62]
DAXX	H3.3	gastric cancer	up	[63]
DAXX	H3.3	pancreatic neuroendocrine tumour	down	[64,65]
CAF-1	H3.3	human breast cancer	up	[66]

## 3. Function of H3.3 in cancer

### 3.1. H3.3 mutations

**Figure 1.**
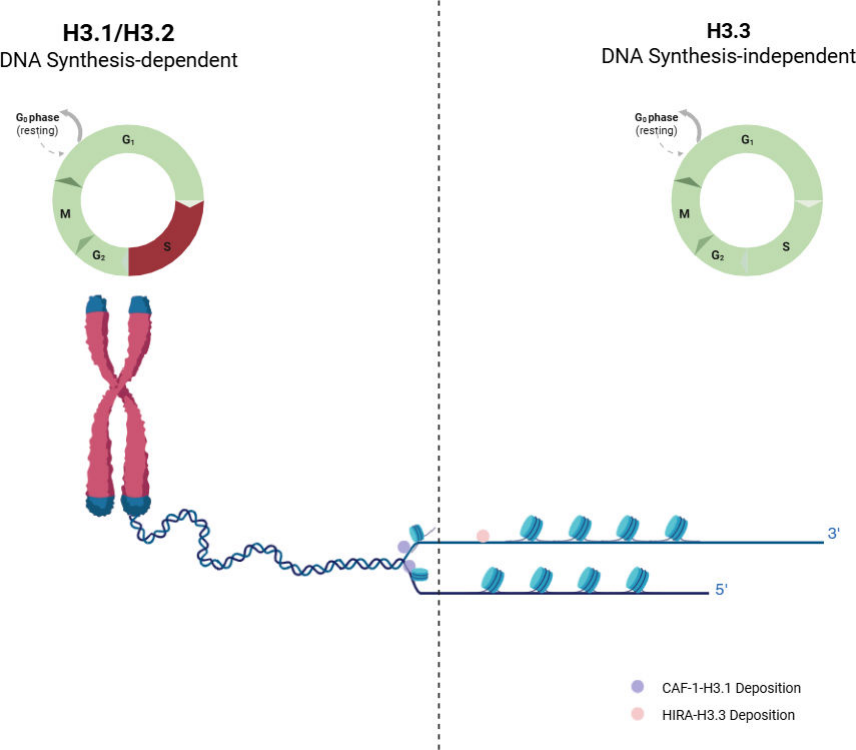
Schematic figure demonstrating H3.1 and H3.3 nuclear incorporation.

The histone variant H3.3 serves as a critical replacement for canonical H3.1 and H3.2 histones, differing by only five amino acid residues at positions 31, 87, 89, 90 and 96 [8,70]. Encoded by the *H3F3A* and *H3F3B* genes located on chromosomes 1 and 17, respectively, H3.3 is expressed constitutively across the cell cycle and in quiescent cells (figure 1) [71]. Its distinct amino acid sequence governs genome-wide deposition patterns, as demonstrated by Goldberg *et al*. [72], who showed that mutating endogenous H3.3b to the H3.2 sequence in mouse embryonic stem cells altered chromatin localization dynamics. While H3.3 is dispensable for viability in *Drosophila*, its loss necessitates compensatory upregulation of canonical H3 genes to preserve chromatin integrity, though its precise role remains indispensable for male germline chromatin condensation, segregation and recombination during meiosis [73,74]. Post-translational modifications of H3.3—including methylation, acetylation, ubiquitination, phosphorylation and neddylation—fine-tune its regulatory functions in chromatin state maintenance and structural organization [75]. Notably, recurrent somatic mutations in H3.3 have emerged as hallmarks of multiple cancers. Seminal studies in 2012 identified high-frequency H3.3 alterations (e.g. K27M, G34R/W/V/L) in paediatric high-grade gliomas, with subsequent discoveries implicating these mutations in chondroblastoma, giant cell tumours of bone, lung cancer and osteosarcoma [26,27,76] (table 3). These heterozygous mutations exert dominant oncogenic effects, driving tumorigenesis through mechanisms linked to chromatin dysregulation, though the precise pathways remain under active investigation. The dysregulation of H3.3 in diverse cancers underscores its central role in maintaining epigenetic fidelity, with somatic mutations acting as potent drivers of oncogenic chromatin remodelling—a paradigm that highlights H3.3 as both a biomarker and therapeutic target in precision oncology.

**Table 3.** H3.3 mutation in cancer.

mutation type	cancer type	references
K27M mutation	paediatric DIPG	[77]
K27M mutation	diffuse brainstem gliomas	[78]
K27M mutation	paediatric glioma	[79]
K27M mutation	acute myeloid leukaemia	[80]
K27M mutation	melanoma	[29]
K27M mutation	posterior fossa ependymomas	[81]
K36M mutation	chondroblastoma tumorigenesis	[82]
K36M mutation	head and neck squamous cell carcinomas	[83]
K36M mutation	paediatric high-grade gliomas	[84]
G34 mutation	osteosarcoma	[82]
G34 mutation	human paediatric glioblastoma	[85]
G34 mutation	neuroblastoma	[86]
G34 mutation	medulloblastoma	[86]
G34 mutation	rhabdomyosarcoma	[86]
G34 mutation	Wilms's tumour	[86]
G34 mutation	prostate cancer	[86]
G34 mutation	lung cancer	[86]
H3.3S31ph mutation	ALT cancer	[87,88]

### 3.2. K27M mutation

The H3.3 K27M mutation, first identified in 2012, arises from recurrent alterations in the *H3F3A* gene of histone variant H3.3, leading to amino acid substitutions at critical residues (K27M and G34R/G34V) within the histone tail [26,27]. This mutation is highly prevalent in paediatric diffuse intrinsic pontine gliomas (DIPGs), where lysine 27 is replaced by methionine (K27M) in H3.3 [77]. The H3K27M mutation drives oncogenic epigenetic reprogramming, characterized by upregulated H4K16ac, reduced H3K9me2 and global depletion of H3K27me3 [89]. Tomita *et al.* [90] demonstrated that H3K27M amplifies platelet-derived growth factor subunit B (PDGFB) expression in Nestin^+^ and Olig2^+^ cells, promoting DIPG tumorigenesis *in vivo*. Furthermore, PRC2—the polycomb repressive complex mediating H3K27 methylation via its catalytic subunit EZH2—is essential for patient-derived DIPG cell proliferation. Pharmacological inhibition of EZH2 suppresses DIPG growth by reactivating the tumour suppressor p16/INK4A [91]. Mechanistically, H3K27M sequesters PRC2 by binding its SET domain, silencing tumour-suppressive loci [92]. While H3K27M also occurs in H3.1, H3.1K27M-bearing cells exhibit significantly lower H3K27me3 levels compared with H3.3K27M counterparts, suggesting variant-specific epigenetic impacts [93]. Live-cell single-molecule tracking revealed that H3.3K27M prolongs EZH2’s chromatin residence time, enhancing its dysregulation without altering chromatin-bound fractions [94]. In murine models, H3.3K27M potentiates neural stem cell self-renewal and collaborates with PDGFRα activation and Trp53 loss to accelerate brainstem gliomagenesis [78]. Beyond gliomas, H3.3K27M has been detected in melanoma, posterior fossa ependymomas and acute myeloid leukaemia (AML) [29,81,95]. In AML, H3K27I/M mutations exacerbate disease progression in RUNX1-RUNX1T1-driven models [80], while in paediatric gliomas, H3K27M upregulates oncogenic cancer-testis antigens like IL13RA2 and VCX3A/B [79]. The H3.3K27M mutation emerges as a central epigenetic driver across malignancies, hijacking chromatin-modifying complexes to fuel oncogenic transcription and stemness—a paradigm underscoring its potential as a therapeutic target and biomarker in precision oncology.

In the last decade, multiple EZH2 inhibitors have been developed, including 3-deazaneplanocin A (DZNep), which inhibits S-adenosylhomocysteine (SAH) hydrolase, causing SAH accumulation. SAH, a byproduct of methyl transfer from S-adenosyl-methionine (SAM), blocks further SAM-mediated methyl transfers [96]. In primary AML cells, DZNep induces cell cycle genes and apoptosis, reduces EZH2 and lowers global H3K27me3 [97]. However, it also lessens other histone methylation marks (H3K4me3, H3K9me1/2/3, H3K36me3, H3K79me3, H3R2me2 and H4K20me3) due to the SAM dependence of other methyltransferases, lacking EZH2 specificity [98,99]. Whether EZH2 acts upon H3.3K27M is still underexplored.

### 3.3. K36M mutation

The H3.3K36M mutation is a key driver of chondroblastoma tumorigenesis, inducing epigenetic reprogramming of the H3K36 methylation landscape and promoting oncogenic hallmarks such as apoptosis resistance, enhanced clonogenicity and impaired cellular differentiation [79,82,100]. A study utilizing parallel sequencing has demonstrated remarkable tumour-type specificity among H3.3 mutations: 95% (73/77) of chondroblastoma cases harboured K36M mutations in *H3F3B*, 92% (49/53) of giant cell tumours of bone exhibited G34W and, in one case, G34L mutations in *H3F3A* [82]. The H3.3K36M mutation has been demonstrated to exert a dominant negative effect on H3K36 methylation, resulting in the depletion of both dimethyl (me2) and trimethyl (me3) modifications [101]. Corroborating this finding, expression of H3.3K36M in HEK 293T and HT1080 cells led to the global reduction of both H3K36me2 and H3K36me3 levels [102]. Baumhoer *et al.* further showed that the H3.3K36M mutation can impact mesenchymal progenitor cells, leading to the development of undifferentiated sarcoma [103]. Specifically, the H3.3K36M mutation led to the inhibition of several H3K36 methyltransferases, including NSD1, NSD2 and NSD3. In the context of human papillomavirus-negative (HPV−) head and neck squamous cell carcinomas (HNSCC), Cavanagh *et al.* confirmed that H3K36M and NSD1 defects both alter H3K36 methylation, thereby impeding cellular differentiation and promoting oncogenesis [83]. Polycomb repressive complex 1 (PRC1) is an important epigenetic regulator that promotes facultative heterochromatin formation and maintains the silencing of PRC1 target genes through two main pathways: compressing chromatin structure and catalysing H2AK119ub1 (a histone) modification. The loss of H3K36 methylation led to the redistribution of PRC1 and the de-repression of its target genes known to block mesenchymal differentiation [104]. Another study demonstrated that H3.3K36M inhibited at least two H3K36 methyltransferases, NSD2 and SETD2, in human chondroblastoma and chondrocytes [100]. BS69/ZMYND11, a specific reader for the modified form of H3.3K36me3, has been suggested as a potential tumour suppressor. This protein is known to regulate pre-mRNA processing by connecting H3.3 lysine 36 trimethylation to chromatin modifications [105]. The K36M substitution in H3.3 prevents K36 methylation, interfering with the binding of H3.3-specific K36me3 readers. Notably, ZMYND11 is crucial for repressing a transcriptional elongation essential for tumour cell growth [105,106]. For instance, the development of paediatric high-grade gliomas often involves hijacking the H3K36me3 pathway, either through K36M mutations or G34R/V/W/L mutations [84]. In a recent study, researchers expressed H3.3K36M in Drosophila and investigated changes in H3 post-translational modifications and gene expression. These findings reveal an unanticipated role for H3K36me2 in maintaining the silent genome and emphasize the non-redundant specificity of individual H3K36 lysine methyltransferases on the chromatin [107]. The H3.3K36M mutation emerges as a master epigenetic disruptor, hijacking methylation-dependent differentiation checkpoints and chromatin regulatory networks to fuel tumorigenesis—a vulnerability that positions H3K36me2/me3 pathways as promising therapeutic targets in chondroblastoma and beyond.

### 3.4. G34 mutation

In osteosarcoma, G34R substitution has been identified in both *H3F3A* and *H3F3B* genes. A recent study demonstrated that RACK7 (ZMYND8) specifically recognizes the H3.3G34R mutation, suppressing MHC class II genes indirectly and directly repressing transport genes such as CIITA [85]. CRISPR-based knock-in correction of the H3.3G34R mutation in paediatric glioblastoma (pGBM) cells significantly reduces RACK7 chromatin binding and inhibits the same gene set as RACK7 knockout in H3.3G34R pGBM cells [85]. G34 mutations are also linked to MYCN upregulation, a potent oncogenic driver in the MYC proto-oncogene family (c-MYC, MYCN, MYCL). MYCN, a 60–63 kDa protein, functions downstream of developmental signalling pathways that regulate growth, proliferation and metabolism in progenitor cells [86,108]. Aberrant MYCN expression is implicated in neuroblastoma, medulloblastoma, rhabdomyosarcoma, Wilms’s tumour, prostate cancer and lung cancer [86]. In paediatric glioblastoma, *H3F3A* G34 mutations elevate MYCN expression via H3K36me3-dependent mechanisms, driving incurable cerebral hemispheric GBMs through MYCN overexpression in neural stem cell compartments [109,110]. Specifically, MYCN is introduced into the stem-cell compartment of the developing forebrain, leading to the development of incurable cerebral hemispheric GBMs [109,110]. Beyond H3K36me3 dysregulation, H3.3G34R mutations alter H3K27me3 patterns and DNA methylation [111,112]. A study that employed HeLa cell lines expressing either wild-type (WT) H3.3, G34L or G34W mutants revealed that these histone H3.3 G34 mutations alter K36 and K27 methylation in *cis* [111]. These mutations can impact the binding of readers specific to K36 or K27 methylation. Furthermore, it was found that G34 mutations inhibited HIRA binding [111]. Recently, Kosuke Funato’s lab established an H3.3G34R mutant glioma model derived from human embryonic stem cells and revealed that mutations in H3.3G34R, ATRX and TP53 have a synergistic effect on alternative RNA splicing events, particularly in the suppression of intron retention. This results in an elevated expression of components of the Notch pathway, notably the human-specific gene family NOTCH2NL [113]. In addition, the clinical treatment of H3.3-G34 mutated glioma still has a serious poor prognosis, including radiotherapy and chemotherapy, surgery and immunotherapy. New combination strategies, such as combining immunotherapy with radiotherapy, have shown some positive results in some pHGG studies [114]. The H3.3G34R mutation drives oncogenesis through epigenetic dysregulation of MYCN, NOTCH signalling and RNA splicing, while its resistance to current therapies underscores the urgent need for targeted approaches leveraging mechanistic insights into its chromatin and transcriptional rewiring.

### 3.5. H3.3 histone chaperones

To date, seven chaperones have been identified for histone H3.3, including FACT, NAP1, ASF1, NASP1, DAXX-ATRX, HIRA and DEK [18]. Among these, DAXX has garnered significant attention in cancer research. DAXX was first discovered in 1997 as a FAS-binding protein and modulator of Jun N-terminal kinase (JNK), which are known to mediate cell death [115]. Later in 2010, DAXX was reported to interact with ATRX and function as an H3.3 chaperone [116–118]. Overexpression of DAXX has been observed in various tumour types, including prostate cancer, ovarian cancer, oral squamous cell carcinoma (OSCC) and gastric cancer [59–63]. Li *et al.* showed that DAXX was highly expressed in OSCC patient tissues compared with normal mucosa tissues [62]. Using lentivirus-mediated shRNA knockdown of DAXX in SAS and SCC25 cell lines, the researchers discovered that suppressing DAXX expression significantly reduced cell proliferation and tumour growth both *in vitro* and *in vivo*. Furthermore, analysis revealed that RNAi-mediated suppression of DAXX led to reduced cyclin D1 mRNA expression as well as overall protein levels in human OSCC cells. Previous studies have established that cyclin D1 expression is dependent on β-catenin/TCF4-mediated transcriptional regulation [119]. This particular study demonstrated that DAXX interacts with TCF4 in OSCC cells, thereby regulating the expression of cyclin D1 [62]. Given the critical role of cyclin D1 in the G1 to S cell cycle transition, the researchers examined the effects of DAXX silencing on cell-cycle progression and observed that DAXX silencing induced cyclin D1-mediated G1 arrest in OSCC cells [62,120,121]. Moreover, Chen *et al.* conducted a comprehensive analysis of DAXX expression and localization (nuclear and cytoplasmic) in 70 pairs of OSCC tissue samples using immunohistochemistry (IHC), DAXX nuclear-to-cytoplasmic ratio (NCR) and survival analysis [122]. Their findings revealed distinct differences in DAXX expression between gastric cancer (GC) tissues and adjacent normal tissues. Specifically, the NCR was significantly higher in GC tissue compared with adjacent normal tissue, and the malignant phenotype in gastric mucosa was strongly correlated with enhanced nuclear accumulation of DAXX [62]. These results suggest that the upregulation of DAXX may serve as a potential diagnostic marker for gastric cancer. Interestingly, a study led by Hoelper *et al.* demonstrated that H3.3 is able to stabilize DAXX and modulate DAXX-associated gene expression independently of its incorporation into the nucleosome [64]. Mutations in the DAXX gene in Pan NETs (pancreatic neuroendocrine tumours) were identified in the folded 4HB and HBD domains, suggesting that loss of H3.3 chaperone may contribute to abnormal chromatin structure, chromosome instability, and epigenetic dysregulation [64,65]. A separate study showed that in addition to DAXX, HIRA was also capable of depositing H3.3 variant throughout the cell cycle [123]. Moreover, the study showed that increased H3.3 is not the sole factor in promoting tumour progression and EMT.

### 3.6. Other enhancing factors

#### 3.6.1. H3.3S31ph

Previous studies have established that CHK1, a histone kinase, phosphorylates H3S10 and T11 [124,125]. A recent investigation demonstrated that CHK1 functions as a novel H3.3S31 kinase and is able to catalyse H3.3S31 phosphorylation *in vitro* [87]. Interestingly, CHK1 is able to promote alternative lengthening of telomeres (ALT) in cancer cells by altering H3.3S31ph dynamics [126]. Specifically, elevated CHK1 activity in ALT cancer cells led to H3.3S31 phosphorylation, which contributed to the maintenance of chromatin integrity and cell survival. Conversely, the absence of H3.3S31 phosphorylation and inhibition of CHK1 activity in ALT cancer cells induced the accumulation of H2AX, a marker for DNA damage-associated chromatin. These findings underscore the pivotal role of CHK1-mediated H3.3S31ph in ALT cancer cell chromatin integrity [87,88]. Recent research has further revealed that specific phosphorylation of H3.3 stimulates the activity of the trans-acetyltransferase p300, indicating that H3.3 can also function as a nucleosome cofactor for p300. Notably, both H3.3S28A and H3.3S28E mutants were able to restore global H3K27ac levels, suggesting that phosphorylation of H3.3S31 may exert a more crucial role than H3S28ph in enhancing p300 activity in mouse embryonic stem cells [127].

#### 3.6.2. MLL5

MLL5 plays key roles in diverse biological processes, specifically in controlling cell cycle progression, maintaining genomic stability and regulating hematopoiesis and spermatogenesis [128]. A study conducted by Gallo *et al.* revealed an inverse correlation between MLL5 protein levels and the expression of *H3F3B*, but not *H3F3A*, in patient-derived GBM primary culture [129]. The study showed that overexpression of MLL5 significantly decreased H3.3 protein levels. Subsequently, the investigators performed next-generation sequencing (ATAC-seq) and demonstrated that MLL5 overexpression shifted open chromatin away from the *H3F3B* transcription start site. Moreover, a significant negative correlation was observed between MLL5 and H3K4me3 in patient-derived GBM primary cultures. However, this inverse correlation was not evident in the parental bulk GBM samples. To further explore whether MLL5 is important for global chromatin organization, the study examined the relationship between MLL5 overexpression and members of the condensin family, proteins crucial for chromosome condensation. Findings from the study indicated that overexpression of MLL5 increased the levels of two condensin family members, NCAPH/BRRN1 and SMC2/hCAP-E [129]. Whether MLL5 overexpression-induced H3.3 reduction is responsible for increased NCAPH/BRRN1 and SMC2/hCAP-E levels remains to be explored.

MLL5 has also been implicated in suppressing the differentiation of self-renewing GBM cells. MLL5 may promote the tumour phenotype by suppressing differentiation, with its downregulation associated with the upregulation of GFAP and TUBB3, and MLL5 overexpression inhibiting immune response-related genes and interferon signalling pathways [129]. The precise methylation of H3K9/27 and H3K4 is crucial for the normal terminal differentiation of retinal cells, yet the role of H3K4me2/3 in activating photoreceptor-specific genes remains elusive [130]. Recently, it has been demonstrated that MLL5 is highly expressed in the mouse retina. Notably, the depletion of MLL5 impairs H3K4 methylation and H3K79 methylation, subsequently compromising CRX-CBP assembly and H3 acetylation on photoreceptor promoters [131].

#### 3.6.3. PTEN

Phosphatase and tensin homologue (PTEN), a ubiquitously expressed tumour suppressor, frequently undergoes mutations in various types of cancers. PTEN serves as a significant negative regulator of class I phosphoinositide 3 kinase (PI3K), AKT and the mechanistic target of rapamycin (mTOR) pathways [132,133]. Benitez *et al.* demonstrated the existence of a chromatin complex comprising PTEN, DAXX and H3.3 [134]. Specifically, the PTEN–DAXX–H3.3 is a chromatin complex that regulates gene transcription in medulloblastomas, endometrial cancer, breast cancer and melanoma [110,135,136]. Using the Human Interactome Map (HiMAP) bioinformatics analysis, the researchers predicted an interaction between PTEN and death domain-associated protein (DAXX). Subsequently, the study confirmed the binding between the unfolded PTEN-hinge domain (amino acids 186-202) and DAXX. DAXX is a prominent histone chaperone protein known to facilitate H3.3 deposition on the chromatin. Collectively, these findings suggest that PTEN may repress targeted gene expression in part by enhancing the levels of H3.3 bound to chromatin [137]. This is also supported by the finding that PTEN regulates oncogene expression by controlling the association and deposition of DAXX-H3.3 on the chromatin [134].

#### 3.6.4. GPR87

GPR87 encodes the G-protein-coupled receptor and plays a crucial role in tumour development and maintenance [138]. A study led by Park *et al.* demonstrated that the overexpression of *H3F3A* increased GPR87 expression by suppressing the inhibitory activity of the GPR87 intronic regulatory DNA element (IRE) and enhancing the invasion ability of lung cancer cells [139]. They demonstrated that H3.3 is able to occupy the GPR87 intronic region, establishing a physical interaction between the transcription start site (TSS) and GPR87 IRE. Furthermore, analysis revealed that H3.3 binding to the GPR87 IRE directly regulates the transcriptional machinery at GPR87. Consequently, the researchers concluded that the combination of *H3F3A* and GPR87 promotes lung cancer progression and migration [139].

## 4. Unifying framework for the oncogenic mechanisms of H3.1 and H3.3

While the distinct associations and mutations of H3.1 and H3.3 across various cancers are well-documented (tables 1–3), integrating these findings reveals fundamental differences and potential convergences in their oncogenic mechanisms. This synthesis highlights how variant-specific dysregulation disrupts chromatin dynamics in a context-dependent manner, influencing tumour type and behaviour. Below, we propose a comparative model summarizing their distinct and overlapping roles, focusing on major cancer categories: gliomas (particularly paediatric high-grade) and carcinomas (e.g. lung, gastric, OSCC).

### 4.1. Distinct mechanisms by cancer context

Recurrent somatic heterozygous mutations (K27M, G34R/V, K36M) act as dominant-negative epigenetic disruptors in H3.3-related paediatric high-grade gliomas. K27M sequesters PRC2, depleting global H3K27me3 while hyperactivating EZH2 locally, silencing tumour suppressors and promoting stemness, with a stronger impact in H3.3 contexts. G34R/V alters H3K36me3 patterns, driving MYCN overexpression, inhibiting MHC-II and disrupting RNA splicing (synergizing with ATRX/TP53 loss to activate NOTCH). K36M inhibits H3K36 methyltransferases, depleting global H3K36me2/me3, redistributing PRC1, de-repressing differentiation-blocking genes and interfering with specific readers. These mutations cause profound epigenetic reprogramming, favouring an undifferentiated state, blocked differentiation, enhanced proliferation and therapy resistance, often occurring early to define disease subgroups.

H3.1-related carcinomas are driven by HIST1 cluster gene dysregulation and altered stoichiometry relative to H3.3. Gene-specific changes in HIST1H3 genes vary by cancer type, impacting local chromatin or pathways like proliferation/apoptosis. A central mechanism is stoichiometric imbalance: aberrant H3.1 polyadenylation leads to excess protein outside S-phase, displacing H3.3 from key regulatory elements and disrupting H3.3-mediated gene regulation critical for homeostasis. CAF-1 overexpression also promotes H3.1 deposition. These processes result in genomic instability, disrupted expression of cancer-associated genes and promotion of malignant phenotypes like proliferation and *in vivo* tumorigenesis, with particular relevance in epithelial cell transformation.

### 4.2. Overlapping and converging pathways

Despite their distinct primary triggers, dysregulation of H3.1 and H3.3 converges on several shared oncogenic pathways, starting with the disruption of chromatin plasticity. Both variants affect nucleosome stability and higher order chromatin structure: H3.3 mutations directly alter post-translational modification landscapes, such as those involving K27me3, K36me2/3 and K4me3, while H3.1 imbalance disrupts the precise incorporation dynamics of H3.3, which is essential for maintaining active chromatin states. This convergence extends to transcriptional dysregulation, as both variants lead to widespread misexpression of genes critical for differentiation, proliferation, apoptosis and immune response. Examples include MYCN upregulation (seen in H3.3-G34R in GBM and potentially impacted by H3.1 imbalance), silencing of tumour suppressors like p16 by H3.3-K27M and activation of oncogenic pathways such as GPR87 by H3.3 in lung cancer.

Another key point of convergence is the impairment of cellular differentiation, a hallmark consequence of both H3.1 and H3.3 dysregulation. H3.3 mutations like K27M and K36M directly block differentiation programmes in neural and mesenchymal lineages, while H3.1 imbalance, potentially by disrupting H3.3 function at lineage-specific enhancers, may similarly hinder terminal differentiation in epithelial cells. Additionally, both variants involve chaperone network dysfunction, with specific chaperones playing roles in their oncogenic effects: CAF-1 overexpression (linked to H3.1) supports replication and proliferation in carcinomas, while dysregulation of DAXX/ATRX (linked to H3.3) through mutations, loss or overexpression disrupts H3.3 deposition, chromatin integrity and gene regulation, as seen in interactions like DAXX-TCF4 affecting cyclin D1. The PTEN-DAXX-H3.3 complex further exemplifies this convergence, where PTEN loss disrupts DAXX/H3.3-mediated gene repression across multiple cancers. Finally, genomic instability arises from both, whether through disrupted nucleosome assembly due to H3.1 imbalance or defective maintenance of heterochromatin from H3.3-K27M, leading to DNA damage and chromosomal instability.

Moving beyond cataloguing associations, this integrated perspective positions H3.1 primarily as a dosage-dependent disruptor of chromatin equilibrium in carcinomas, often through stoichiometric imbalance with H3.3. In contrast, H3.3 acts as a direct epigenetic hijacker via dominant-negative mutations in specific developmental cancers like paediatric glioma. Their convergence on disrupting chromatin plasticity, transcriptional fidelity and differentiation highlights the centrality of histone variant dynamics in oncogenesis. Future research must delineate the spatiotemporal dynamics of H3.1/H3.3 deposition and their interplay with specific enhancer-promoter landscapes across different cancer types to fully exploit these mechanisms therapeutically.

## 5. Future directions

The study of histone variants H3.1 and H3.3 in cancer has revealed their distinct and overlapping roles in chromatin remodelling and transcriptional regulation. However, critical gaps remain in understanding their precise mechanisms and therapeutic potential. Future research should leverage advanced technologies to resolve variant-specific effects and explore novel treatment strategies. Single-cell chromatin profiling, such as single-cell ATAC-seq (scATAC-seq) and single-cell RNA-seq (scRNA-seq), could elucidate the heterogeneity of H3.1 and H3.3 deposition across individual tumour cells. These techniques would reveal how variant-specific alterations influence chromatin accessibility and gene expression in subpopulations of cancer cells, particularly in tumours with mixed epigenetic states. Additionally, integrating single-cell multi-omics (e.g. scATAC-seq with scRNA-seq) could uncover the direct transcriptional consequences of H3.1 and H3.3 dysregulation, identifying shared and unique downstream pathways. The distinct mechanisms of H3.1 and H3.3 dysregulation suggest that variant-specific therapies may be necessary. For H3.3-mutant tumours (e.g. paediatric gliomas), combining epigenetic drugs (e.g. EZH2 inhibitors for K27M or NSD inhibitors for K36M) with immunotherapy could enhance efficacy. The global hypomethylation caused by H3.3K36M may increase tumour immunogenicity, making these tumours responsive to immune checkpoint blockade. Conversely, H3.1-driven carcinomas might benefit from targeting histone chaperones like CAF-1 to restore H3.3 deposition, alongside radiotherapy to exploit resultant genomic instability. Preclinical studies using patient-derived xenografts or organoids could test these combinations, prioritizing agents that synergize with existing standards of care.

A key unanswered question is whether H3.1 and H3.3 alterations converge on shared oncogenic pathways despite their divergent upstream mechanisms. Systems biology approaches, such as network analysis of transcriptomic data from H3.1- and H3.3-altered tumours, could identify common hubs (e.g. cell cycle regulators or differentiation blockers). For example, both variants may dysregulate Polycomb repressive complexes (PRC1/2) or MYC networks, suggesting broad therapeutic avenues. Functional screens (e.g. CRISPR knockout or drug libraries) in variant-specific models could validate these hubs and reveal synthetic lethal interactions.

## 6. Conclusion

In this review, we summarized the functions and expression patterns of canonical histone H3.1 and histone variant H3.3 in different cancers. While our knowledge of H3.1 and H3.3 functions has advanced significantly, the dynamic relationship between canonical histones and their variants remains understudied. Future studies may consider exploring similar downstream genes affected by both H3.1 and H3.3, and whether the related signalling pathways are affected in the same manner in different cancer types.

## Data Availability

This article has no additional data.
